# Unveiling a Dermatological Rarity: The Enigma of Vulvar Intraepithelial Neoplasia Grade III (HSIL) and the Role of p53 in Its Development

**DOI:** 10.3390/biomedicines12081799

**Published:** 2024-08-08

**Authors:** Piotr Brzeziński, Igor Feszak, Beatriz Di Martino Ortiz, Sylwia Feszak, Piotr Kawczak, Tomasz Bączek

**Affiliations:** 1 Institute of Health Sciences, Pomeranian University in Słupsk, 76-200 Słupsk, Poland; piotr.brzezinski@upsl.edu.pl (P.B.); igorfeszak@gmail.com (I.F.); 2 Dermatology Department, Clinicas Hospital, Faculty of Medical Sciences, National University of Ausunción, San Lorenzo 111421, Paraguay; beatrizdimartino@gmail.com; 3 Department of Genetics and Pathology, International Hereditary Cancer Center, Pomeranian Medical University, ul. Unii Lubelskiej 1, 71-252 Szczecin, Poland; sylmoraw@gmail.com; 4 Department of Pharmaceutical Chemistry, Faculty of Pharmacy, Medical University of Gdańsk, 80-416 Gdańsk, Poland; tomasz.baczek@gumed.edu.pl; 5 Department of Nursing and Medical Rescue, Institute of Health Sciences, Pomeranian University in Słupsk, 76-200 Słupsk, Poland

**Keywords:** vulvar cancer, differentiated vulvar intraepithelial neoplasia, p53, p16, HPV

## Abstract

Vulvar intraepithelial neoplasia, also known as VIN, is a non-invasive squamous lesion and precursor of squamous cell carcinoma (SCC) of the vulva. There is no screening test for vulvar intraepithelial neoplasia. Diagnosis of VIN is made clinically and confirmed with a biopsy. We describe a 66-year-old woman with a condyloma-like tumour located in the skin on the vestibule of the vagina. A biopsy sample was taken from the nodule. The definitive diagnosis is supported by the histological examination (VIN III) and immunohistochemical examination of p16(+), p53(+), and a few cell nuclei. The case provides information on the importance of multidisciplinary cooperation. Lifelong surveillance is essential since the resection of individual lesions does not guarantee the prevention of invasive cancer.

## 1. Introduction

Vulvar squamous intraepithelial lesions (SILs), which are also known under a previous name of vulvar intraepithelial neoplasia (VIN), are a group of precancerous conditions of the vulva. Vulvar SIL terminology and classification systems have changed significantly over the years. The newest classification was published in 2015 by the International Society for the Study of Vulvovaginal Disease [1]. However, supporting studies on this topic may have used previous editions of this classification system (Table 1) [1,2,3].

**Figure 1:**
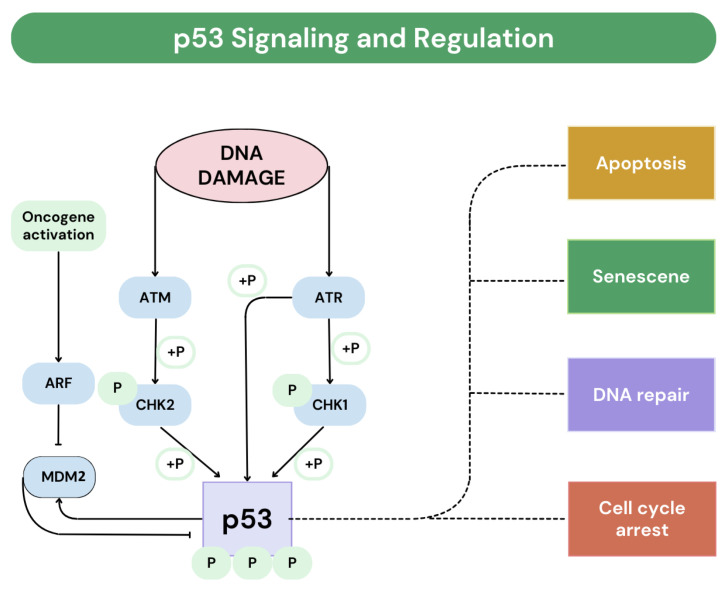
The p53 protein signalling and regulation; ATM (ataxia telangiectasia mutated); ATR (ataxia telangiectasia and Rad3-related); CHK1 (Checkpoint Kinase 1); CHK2 (Checkpoint Kinase 2); MDM2 (E3 ubiquitin protein ligase); ARF (Alternate Reading Frame); +P (Phosphorylation), based on Ref. [7].

There are two known pathways for the development of vulvar squamous cell carcinoma: an HPV (human papillomavirus)-dependent pathway characterized by the over-expression of the p16 protein, and an HPV-independent pathway associated with lichen sclerosus, characterized by mutations in the p53 gene [4]. The protein p53 encoded by the TP53 gene, also known as “the guardian of the genome”, is a key tumour suppressor protein that regulates the cell cycle and prevents cancer. Physiologically, p53 levels are kept low through degradation by MDM2, a ubiquitin ligase. In response to cellular stress or DNA damage, p53 becomes stabilized and activated, leading to cell cycle arrest, DNA repair, or apoptosis [5]. Mutations in the TP53 often lead to (1) disrupted cell cycle control—mutated p53 fails to stop damaged cells from dividing uncontrollably; (2) increased invasiveness and metastasis—mutations can activate pathways that enhance cell migration and invasiveness, promoting metastasis; and (3) interactions with other proteins—mutant p53 can bind to proteins like p63 and p73, altering their functions and increasing the invasive and metastatic capabilities of cancer cells [6,7,8,9]. The protein p16 is encoded by the CDKN2A gene. It is a protein that inhibits cell division by decelerating the transition of the cell cycle from the G1 phase to the S phase, thus serving as a tumour suppressor. A deletion in this gene, which is the loss of a part of the DNA sequence during replication, can lead to insufficient or defective p16. This deficiency speeds up the cell cycle, contributing to the development of tumour transformation [10,11]. The p53 signalling pathway activates in response to cellular stress or DNA damage. When activated, p53 acts as a transcription factor, regulating genes responsible for cell cycle arrest, DNA repair, apoptosis, and cellular senescence (Figure 1). These processes maintain genomic stability and prevent tumour formation. Cellular stress activates p53 by disrupting its interaction with MDM2 (E3 ubiquitin protein ligase), a protein that represses p53 transcriptional activity. This release allows p53 to bind to DNA and promote the transcription of genes, leading to cell cycle arrest or apoptosis. Cell cycle arrest provides time for DNA repair, while apoptosis removes damaged cells, preventing the spread of mutations. p53 plays a significant role in cellular senescence by halting cell division, which is important in aging and age-related diseases. Additionally, p53 regulates cell proliferation, enabling cell division under normal conditions by inhibiting senescence in response to growth signals. The p53 protein is tightly regulated to ensure proper cellular function. MDM2 negatively regulates p53 by inhibiting its activity and promoting its degradation. Cellular stress or DNA damage disrupts the p53-MDM2 interaction, stabilizing and activating p53. Post-translational modifications such as acetylation, methylation, and phosphorylation further influence p53 by regulating its stability, DNA binding, interactions with co-regulators, and target gene specificity. Other proteins and pathways, such as ATM (ataxia telangiectasia mutated) and ATR (ataxia telangiectasia and Rad3-related), also regulate p53 by responding to cellular stress signals and activating or stabilizing p53 [7,12].

The 2015 ISSVD terminology (Table 1) for vulvar SILs is LSIL: low-grade squamous intraepithelial lesion (vulvar LSIL, flat condyloma, or human papillomavirus effect). These were previously referred to as vulvar intraepithelial neoplasia (VIN). HSIL, high-grade squamous intraepithelial lesions (vulvar HSIL and VIN usual type), have previously been referred to as VIN 2 and VIN 3. Lesions known as dVIN, differentiated VIN, include lesions that are not associated with HPV but are associated with vulvar dermatoses, mainly lichen sclerosus, and were previously referred to as the VIN simplex type. At the very beginning, it is worth mentioning that there are no routine screening methods for vulvar SIL or vulvar cancer. The diagnosis of SIL is based on the histological findings observed in the biopsy material. Suitable biopsy sites are identified primarily through physical examination and colposcopy. LSILs are equivalent to anogenital warts, should not be considered potentially cancerous, and often do not need treatment. The exception is LSILs that show clinical symptoms, and such lesions should be treated until symptoms resolve. The main goal of HSIL treatment is to prevent the progression of squamous cell carcinoma and alleviate symptoms while maintaining the normal anatomy and function of the vulva [13]. Management options for HSIL include excision, ablation therapy, and topical treatment. Treatment should depend on the degree of concern about invasive disease based on examination and biopsy results, prior treatment history, and lesion location.

## 2. Case Report

**Figure 2:**
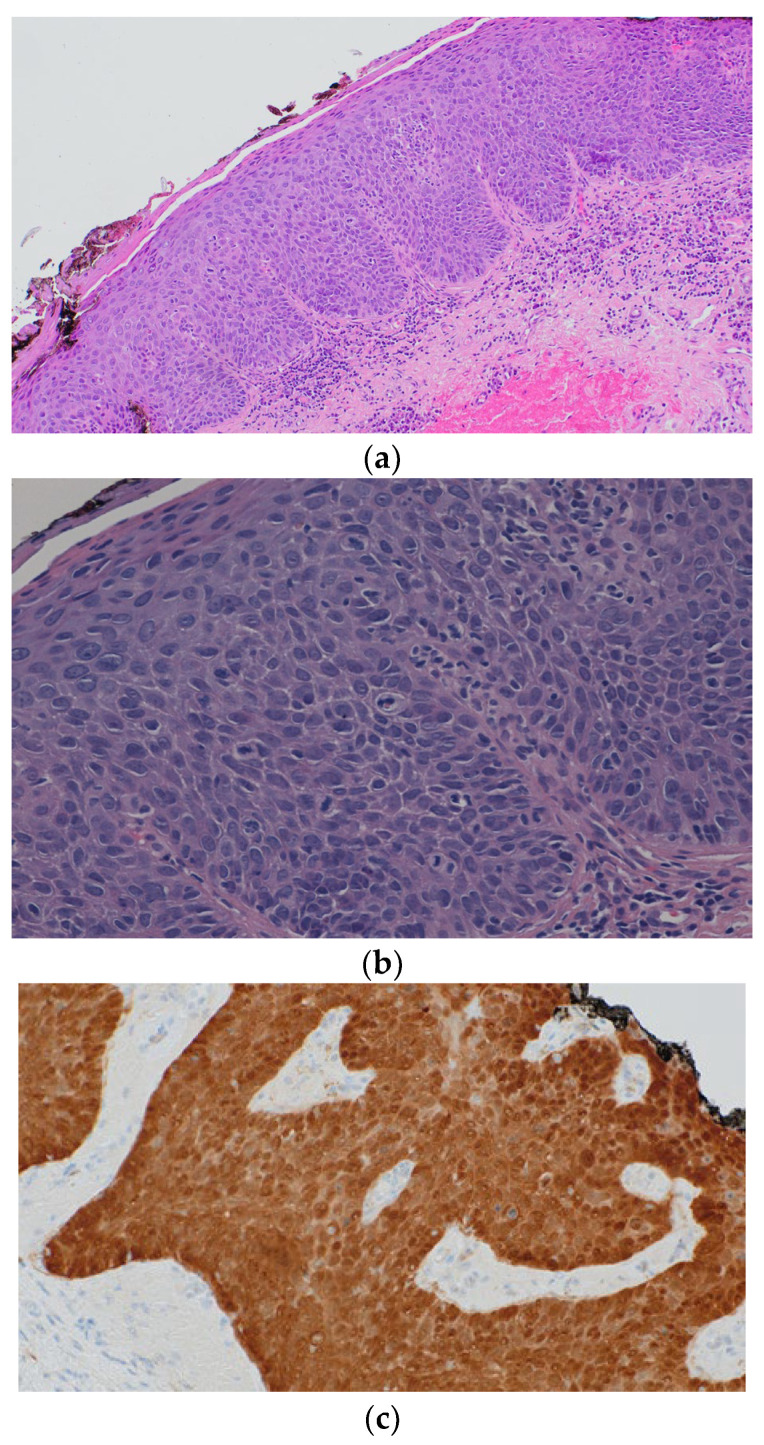
Histological findings in the patient’s biopsy sample: (**a**) epidermal acanthosis, agranulocytosis, and parakeratosis; (**b**) epidermal keratinocytes show loss of cell maturation and polarity that affects the full thickness of the epithelium, the basal membrane is not exceeded, and there are images of atypical mitosis typically arrested in metaphase; (**c**) positive p16 in intraepidermal atypical cells.

A 66-year-old female, in 2022, during a routine follow-up, presented with a nodule in the vulval vestibule without associated complaints (pain, pruritus). The nodule, measuring 6 by 5 mm, was in the lower left part of the vulval vestibule around Hart’s lines. A biopsy of the nodule was performed in the dermatology department. The histological and immunohistochemical examination of the punch biopsy showed p16(+), p53(+), and few cell nuclei (Figure 2). p53 IHC was performed using anti-human p53 protein. Nuclear p53 staining was assessed quantitatively and qualitatively. Staining intensity was divided into no staining (0), weak (1+), intermediate (2+), and strong (3+).

The p16 antibody was used for the examination. Nuclear and cytoplasmic staining were assessed. Staining intensity was classified as no staining (0), weak (1+), intermediate (2+), and strong (3+).

Subsequently, the patient was transferred to the gynaecology department. Staging CT and MRI scans showed asymmetrical thickening of the vulva but no pelvic adenopathies or distant metastases. The patient underwent a radical vaginectomy with urethrectomy and inguinal sentinel node procedure. The final pathology report showed two tumour-free sentinel lymph nodes. All section margins were negative. This was followed by periodic genital examinations and a yearly CT scan of the thorax and abdomen.

There were no relapses of the disease during a year and a half of follow-up.

## 3. Discussion

LSILs are characterised by the mild skin symptoms of an HPV infection, which are usually limited and disappear within one to two years. These lesions are usually not considered potentially cancerous; the exception is condyloma acuminata (genital warts), which, if untreated, may become cancerous. HSILs, on the other hand, are considered intraepithelial neoplasms and are estimated to be associated with approximately 20% of vulvar cancers [14]. Differentiated VIN (dVIN) is associated with the highest risk of progression to SCC of all VINs. This risk is estimated to be as high as 33%. Research shows that dVIN is the precursor to approximately 80% of keratinizing vulvar SCCs [14,15].

Analysing the frequency of p53 and p16 mutations and their expression across different types of vulvar lesions provides valuable diagnostic and prognostic information. In the study conducted by Grapsa et al. (2014) [16], in LSIL cases, no positive results were observed for p53 expression, meaning all 61 cases (100%) were negative. For p16, 44.3% (27 cases) showed positive expression, while 55.7% (34 cases) were negative. In HSIL samples, 36.4% (four cases) showed positive p53 expression, while 63.6% (seven cases) were negative. The expression of p16 in these samples was significantly higher, with 90.9% (ten cases) showing positive expression and only 9.1% (one case) showing negative expression. Overall, for all 92 cases (total), 4.3% (4 cases) showed positive p53 expression, while 95.7% (88 cases) were negative. For p16, 40.2% (37 cases) showed positive expression, while 59.8% (55 cases) were negative. These results suggest that p53 expression is rare in LSIL but may be more common in HSIL. On the other hand, p16 expression is significantly higher in HSIL, confirming its role as a biomarker of HPV-induced malignant transformation (Table 2) [16].

In addition to p16 and p53, Ki-67 labelling can provide valuable information [13,16].

Authors from the Netherlands showed a positive result of p16INK4a blocking in 99.0% of cases and an increase in Ki-67 in ≥2/3 of the epithelium in 93.6% [17].

The clinical picture of SIL may be asymptomatic or manifest with pruritus of the vulva, which is the most common symptom among patients, as well as burning and pain of the vulva, in addition to dysuria. In a meta-analysis of over 3300 VIN 3 patients, 64% of patients reported pruritus or pain in the vulva area [18]. Approximately 40% of patients with SIL do not experience symptoms related to vulvar lesions. The initial diagnosis is made during a routine gynaecological examination or colposcopy due to abnormal cervical cytology results. The main risk factors for HSIL are human papillomavirus, smoking, and immunodeficiency. Most cases of SIL are related to HPV infection. Transmission routes include sexual contact, including genital, anal, or oral contact with the vulva. Vaginal intercourse alone is not required for HPV transmission. In research studies, almost 90% of VIN cases test positive for HPV [19]. LSIL is most often associated with HPV subtypes 6 and 11, although this does not exclude the high-risk types of HPV 16, 18, and 31, which are more characteristic of HSIL [14]. It is worth noting that HPV vaccines are active against HPV 6, 11, 16, and 18 (quadrivalent vaccine) and additionally against HPV 31, 33, 45, 52, and 58 (nine valent), which significantly reduces the risk of vulvar SIL [20,21,22]. In addition, consistent cigarette smoking is associated with the development of vulvar SIL [17,23,24]. One study found a significant association between VIN and cigarette smoking by comparing a group of patients with VIN, in which the majority smoked cigarettes, to a group of non-smoking women of the same age with non-cancer diseases of the vulva [25]. Premenopausal patients are more likely to have HPV-related vulvar HSIL, while postmenopausal patients are more likely to have non-HPV-related vulvar SIL. The main risk factor for dVIN is a coexisting vulvar dermatosis such as lichen sclerosus. The cumulative incidence of vulvar SCC in patients with lichen sclerosus is 6.7%. The risk is higher in patients with lichen sclerosus and VIN (18.8%) compared to patients with lichen sclerosus alone (2.8%) [26].

Medical history examination should include questions about the risk factors and symptoms associated with SIL. Patients should be asked about their previous medical history, genital warts, vulvar cancer, and other lower genital tract cancers (especially cervical cancer). The medical history should also include questions about smoking, immunodeficiency, and HPV vaccination. A physical examination should be performed, using the palpation of the vulva and groin to check for lesions, coloured lesions, lumps, or sores. There is no pathognomonic picture of SIL. Most SILs of the vulva are located in the hairless part of the vulva, and these are multifocal [27]. The lesions are often raised or verrucous and white. Other vulvar lesions, like lichen planus, lichen sclerosus, and condyloma latum, can mimic VIN. Any vulvar lesion found during examination that is not benign requires a biopsy. If a lesion is treated without a biopsy, does not resolve completely, or is refractory to empirical treatment, a biopsy should be considered for a definitive diagnosis. If a lesion cannot be detected by colposcopy, a biopsy of the symptomatic vulva should be performed.

Histologically, LSIL is characterized by cell atypia, increased mitotic activity in the basal or sub-basal epithelium, and squamous maturation in the upper two-thirds of the epithelium. HSIL shows the impaired maturation of the middle- or upper-third to full-thickness squamous epithelium. HSIL can be divided into three subtypes: (1) The basaloid type consists of atypical, immature basal cells with numerous mitotic figures and enlarged, hyperchromatic nuclei. It has a thickened epithelium with a relatively flat, smooth surface. (2) The warty (condylomatous) type has a striated or spiky surface, which gives the appearance of condylomas. Significant cell proliferation with numerous mitotic figures and abnormal maturation is observed. Confluent or multifocal lesions occur in up to two-thirds of patients with HSIL. (3) The differentiated type (simplex) has cells that are confined to the basal and sub-basal levels with or without atypia above the basal or sub-basal layers. The epithelium is parakeratotic, thickened with elongated and anastomotic rete ridges. p53 is often mis-expressed. Distinguishing dVIN from reactive squamous cell proliferation may prove to be complicated [28].

p53 immunohistochemistry serves as a prognostic marker for favourable and unfavourable outcomes [29].

Histopathology is the standard of reference for the diagnosis of SIL. However, studies have shown considerable variability in the histological classification of CIN [30]. The rate of CIN2 diagnoses continued to vary considerably between laboratories. It is more likely that this difference in the rate of CIN2 diagnoses is due to variability in the classification of histopathologists between CIN2 and CIN1, as well as CIN2 and CIN3 diagnoses.

In the differential diagnosis of SIL, we should consider candidiasis dermatitis, molluscum contagiosum, lichen sclerosus, lichen planus, lichen simplex chronicus, psoriasis, and herpes simplex virus infections, and other nonspecific nodules such as syringocystadenoma papilliferum, syringoma, and angiomyxoma [29,31,32,33]. Vaginal candidiasis is an infection of the oestrogenised vagina that can spread to the labia minora, labia majora, and perianal area. Candidiasis of the cervix and endometrium does not occur. The dominant species found in asymptomatic patients, premenopausal women, and pregnant women is Candida albicans [34]. The predominant symptom is itching, which is not unique to vaginal candidiasis. Other common symptoms include redness, burning, pain, dysuria, and dyspareunia. The gold standard in the diagnosis is based on finding hyphae or pseudohyphae in direct microscopy in conjunction with the patient’s clinical picture [34]. For ambiguous results, diagnostics should be extended to biological culture. Serological tests may complement the primary diagnosis of vaginal candidiasis but are not mandatory. Lichen planus (LP) is a chronic inflammatory disease with significant cosmetic and functional impact. If left untreated, LP can lead to scarring and nail loss. The pathogenesis of LP is currently considered unclear, but disturbances in the Toll-like receptor (TLR) function probably play a role. LP usually presents as a papulosquamous lesion of varying size, often described by the so-called “six P” (pruritic, purple, planar, polygonal, papules, and plaques) [35]. LP has the characteristic “Wickham’s mesh”. Diagnosis is based mainly on the clinical picture of the lesion, sometimes with the help of dermatoscopy. Often, a biopsy specimen of the skin is helpful for confirming a clinical diagnosis. Chronic lichen vulgaris is characterized by erythematous, scaly lesions that form plaques. Often, such changes are accompanied by traces of abrasions. Sudden hyperpigmentation and hypopigmentation may also occur. The presence of lichenization and excoriation is usually sufficient for making a clinical diagnosis [36]. Sometimes, the only symptom may be erythema. This is often the case when the disease has just begun. Notably, the absence of atopy does not interfere with diagnosing LSC. Eosinophilia and elevated IgE levels have low specificity. Biopsies taken from lesions during the period of scratching and rubbing are often of little diagnostic value due to the so-called background noise effect [37]. Distinguishing LSC from psoriasis is particularly difficult due to the similarity between the two. The main question in diagnosing LCS should be whether LSC is a primary lesion or secondary to another underlying disease. Psoriasis is a non-infectious, chronic, systemic inflammatory disease characterized by specific skin lesions resulting from the hyperkeratosis of the epidermis. Psoriasis has three principal histological features: epidermal hyperplasia, prominent blood vessels, and an inflammatory infiltrate of leucocytes. The hyperplastic epidermal changes are associated with the loss of the granular cell layer, parakeratosis, the elongation of rete ridges, and the presence of Munro microabscesses [38]. A characteristic symptom is specific skin changes from a disturbed regeneration process of the epidermis. In the initial stage, there is a primary eruption, a demarcated, red-brown papule with an exfoliating surface. Fully developed changes, the plaques are more extensive and covered with tightly adherent silvery scales (psoriatic plaques). When the scale is scraped off, one can observe a shiny surface (stearin candle symptom) and tiny droplets of blood (Auspitz symptom) in its location. Although most HSV genital infections present as asymptomatic and atypical, they may be characterised by the bilateral clusters of erythematous papules and vesicles on the external genitalia. HSV lesions can also be observed in the perianal region. Symptoms that patients may experience are pain, itching, burning, and dysuria. Primary HSV-1 infection cannot be distinguished from primary HSV-2 infection by standard blood testing alone [39]. For this purpose, serological tests should be used. Over time, the lesions develop into vesicles and pustules, which may coalesce into ulcers without forming pustules or crusts. Preliminary clinical diagnosis should prompt the initiation of antiviral treatment before the laboratory confirmation of an HSV infection [37]. One study showed that 25% of people with symptoms of first genital herpes infection had serological evidence of a history of HSV-2 infection at the time of presentation, suggesting that the initial infection was asymptomatic [38,40].

Thuijs et al. found that HPV-independent VIN with p53 mutant IHC or nondifferentiated morphology has distinctive pathological and behavioural features. Both subtypes are highly aggressive and require more careful monitoring after surgery. The examination of p16INK4a and p53 IHC in each newly diagnosed VIN lesion is highly recommended [17].

Before starting treatment, a thorough colposcopic examination with a biopsy to exclude invasive disease is mandatory. The treatment method should depend on the degree of invasiveness of the disease, which is based on the examination results, histopathological examinations, and the location of the lesion. First-line HSIL treatment, requires the excision of the lesion through a wide local excision, defined as the excision of a single lesion with a margin of 1 cm. A wide local excision should produce satisfactory cosmetic results. One study with a cohort of 303 patients found that the surgical excision of a lesion is associated with a significantly lower risk of recurrence (26.4%) compared to the method of laser ablation (41.9%) [41]. An additional advantage of this method is that it provides both treatment and diagnostic material, which is particularly important if we take into account the risk of the frequent occurrence of this disease during the latent phase [42,43]. If we suspect that the excision of the lesion would result in negative structural or functional consequences, e.g., in the case of the lesion adjacent to the anus or urethra, the patient should be referred to a gynaecologist. If the patient expects complete lesion removal with a highly satisfactory cosmetic effect, we can consider ablation therapy with carbon dioxide or an argon beam coagulator. For patients with risk factors for disease recurrence, such as heavy smoking and immunodeficiency, it is worth considering ablative or local therapy to protect the patient from significant structural changes and loss of function in this area. The goal of ablative therapy is to treat the entire area of intraepithelial abnormality. In these cases, tissue is ablated rather than resected; therefore, the coexistence of invasive cancer must be carefully ruled out by the casual use of colposcopically targeted biopsies before surgery. For patients with clitoral lesions, topical imiquimod treatment may be considered if the patient commits to extended treatment, potentially lasting up to 6 months. Imiquimod is a topical immunomodulator often used as the initial treatment for relapsing vulvar HSIL. Imiquimod is applied topically, not to the entire vulva [44]. Topical treatment with fluorouracil has a limited role in the primary therapy of HSIL because it is associated with common side effects such as burning, pain, oedema, and ulceration.

## 4. Conclusions

The current article underscores the critical need for the accurate diagnosis of vulvar squamous intraepithelial lesions and vulvar cancer, emphasising that due to the absence of routine screening methods, the histological examination of biopsy material remains essential. Regular gynaecological exams and thorough colposcopic evaluations are vital for early detection. Biomarkers such as p16 and p53 provide valuable diagnostic and prognostic insights, with p16 strongly linked to HSIL, confirming HPV’s role in malignant transformation. Conversely, p53, although rare in LSIL, is more common in HSIL and suggests potential mutations. The management of HSIL should be tailored to the disease’s severity, including options like excision, ablation therapy, and topical treatment, considering the lesion’s location and previous treatment history. The article highlights the necessity of multidisciplinary cooperation among gynaecologists, dermatologists, and oncologists due to the often-asymptomatic nature of HSIL and its low symptom specificity. External factors such as cigarette smoking and immunodeficiency are linked to SIL development, and the significant role of HPV vaccinations in reducing vulvar SIL risk is emphasized. Long-term patient monitoring is crucial, with regular follow-up exams, to detect any recurrences or disease progression early.

## Figures and Tables

**Table 1:** HSIL: high-grade squamous intraepithelial lesion; LSIL: low-grade squamous intraepithelial lesion; N/A: not applicable; ISSVD: The International Society for the Vulvovaginal Disease; LAST: Lower Anogenital Squamous Terminology.

1986 ISSVD	2004 ISSVD	2012 LAST	2015 ISSVD
VIN 1 (mild dysplasia)	Condyloma	LSIL	LSIL of the vulva (flat condyloma, vulvar LSIL, HPV effect)
VIN 2 (moderate dysplasia)	VIN, usual type		
	VIN, warty type VIN, basaloid type VIN, mixed type	HSIL	HSIL (vulvar HSIL, VIN usual type)
VIN 3 (severe dysplasia) VIN 3 (carcinoma in situ)			
	VIN differentiated	N/A	Differentiated VIN
		
Differentiated VIN			
		

**Table 2:** Frequency of p53 and p16 expression in LSIL and HSIL; LSIL: low-grade squamous intraepithelial lesion; HSIL: high-grade squamous intraepithelial lesion according to Ref. [16].

Type	p53(+) % (*n*)	p53(−) % (*n*)	p16(+) % (*n*)	p16(−)% (*n*)
LSIL	0% (0)	100% (61)	44.3% (27)	55.7% (34)
HSIL	36.4% (4)	63.6% (7)	90.9% (10)	9.1% (1)
Total	4.3% (4)	95.7% (88)	40.2% (37)	59.8% (55)

## Data Availability

The data presented in this study are available on request from the corresponding author. The data are not publicly available due to restrictions concerning privacy and ethical reasons.
